# Plasmon-Coupled Whispering Gallery Modes on Nanodisk Arrays for Signal Enhancements

**DOI:** 10.1038/s41598-017-12053-8

**Published:** 2017-09-15

**Authors:** Tae Young Kang, Wonju Lee, Heesang Ahn, Dong-Myeong Shin, Chang-Seok Kim, Jin-Woo Oh, Donghyun Kim, Kyujung Kim

**Affiliations:** 1 Pusan National University, Department of Cogno-Mechatronics Engineering, Busan, 46241 Republic of Korea; 2 Yonsei University, School of Electrical and Electronic Engineering, Seoul, 03722 Republic of Korea; 3 Pusan National University, Research Center for Energy Convergence Technology, Busan, 46241 Republic of Korea; 4 Pusan National University, Department of Optics and Mechatronics Engineering, Busan, 46241 Republic of Korea; 5 Pusan National University, Department of Nano Energy Engineering, Busan, 46241 Republic of Korea

## Abstract

Metallic nanostructures including single and double nanodisks are successfully used to enhance the localized electric field in vicinity of microcavity in whispering gallery mode (WGM) sensor. We demonstrate numerical calculations of plasmonic coupling of WGMs to single and double nanodisk arrays on a planar substrate. We then experimentally confirmed that the resonance wavelength of WGM sensor was dramatically shifted by adoption of single and double nanodisks on the surface of microcavity in the WGM sensor. Thus, our approach provides the tunable sensitivity of WGM sensor, and has a great potential to be used in numerous areas where the single biomolecule, protein-protein folding and biomolecular interactions are involved.

## Introduction

A Whispering gallery mode (WGM) biosensor has been a well-defined emerging technique for label-free detection of biomolecules, their conformations and interactions^1–7^. The sensitivity of WGM biosensor relies on the use of high quality factor (Q-factor) photonic resonances which occur, for example, when light interferes constructively inside a ~50–400 µm glass cavity trapped by total internal reflection. The binding of target entities to the cavity surface is detected by monitoring the spectral shifts of resonance wavelength^8–13^. A red-shift of the resonance wavelength occurs upon analytical binding since bound molecule produces slight increase in optical path length. For this reason, the WGM biosensor has been employed for ultrasensitive detection with a detection limit down to the single virus and possibly the single molecular level^6^. However, the sensor still requires extrasensitivity to clearly detect single molecular interactions such as a conformation change of single biomolecule, single protein-protein folding and a single antibodyantigen interaction in real-time. An approach based on the field intensity enhancements in WGM biosensing is one of the important issue for achieving ultra-high sensitivity^14–17^. From the first-order perturbation approximation, these resonance wavelength shifts ∆λr are evaluated according to

1 $$\frac{{\rm{\Delta }}{\lambda }_{r}}{{\lambda }_{r}}\cong \frac{\alpha /{\varepsilon }_{0}{|{\boldsymbol{E}}({{\boldsymbol{r}}}_{{\boldsymbol{v}}})|}^{2}}{2\int {\varepsilon }_{r}({\boldsymbol{r}}){|{\boldsymbol{E}}({\boldsymbol{r}})|}^{2}{\boldsymbol{dV}}}$$

where α is the polarizability of the molecule in excess to the surrounding medium, r_v_ indicates the molecular binding site, E is the WGM electric field strength, λ_r_ the nominal laser wavelength, ε the permittivity of the cavity^18^. As can be seen from equation (1), the resonance wavelength shift increases in proportion to the local electric-field intensity |E(r_v_)|^2^ that a biomolecule encounters at its binding site. Any mechanism that can increase the field intensity at the binding site without significantly degrading the Q-factor of the WGM resonance will therefore produce an enhancement in the wavelength shift, thereby enhancing the detection sensitivity.

Plasmon based concepts have been suggested with varied nanostructures which create sites of high field intensities (hotspots)^19–25^. A drawback of this previous demonstration of a sensitivity-enhanced WGM biosensor, however, arises from the random nature of the nanoparticle layer, since consequently the location and intensity of field enhancements cannot be estimated and controlled. Furthermore, because the exact structure of the random layer at the WGM coupling region is uncertain, it is difficult to utilize numerical calculations to make predictions which could then be subjected to experimental verification. Especially, metallic nanostructures on chip provide ideal platforms to study the effect of plasmonic field localization in WGM biosensing. Moreover, such a planar platform would allow precise control of optical field localization in the vicinity of the nanostructures^26,27^. There have been lots of previous studies about localized plasmonic fields with nanostructures on chip^28–31^. These efforts give significant potentials to enhance sensitivity in Raman, extraordinary transmission or surface plasmon resonance sensing^32–34^.

In this paper, we investigated the plasmon coupling effect using gold nanodisk arrays on chips for a WGM biosensor. Initially, we designed two different models to analyze (or, for analysis of) plasmon coupling by existence of nanodisk arrays on chips. Single and double nanodisk arrays were modeled and perturbations of a WGM resonance wavelength were numerically calculated by coupling of nanodisk arrays. Furthermore, the near-fields distributions by plasmon coupling at WGM resonance wavelengths were simulated. Consequently, we experimentally verified the plasmon coupling effects using single and double nanodisk arrays which were fabricated on chip by electron beam (E-beam) lithography. We experimentally estimate and compare the energy fraction of the WGMs fields that localized at the single and double nanodisk arrays.

## Results and Discussion

### Concept of the plasmon-coupled WGM sensor

**Figure 1:**
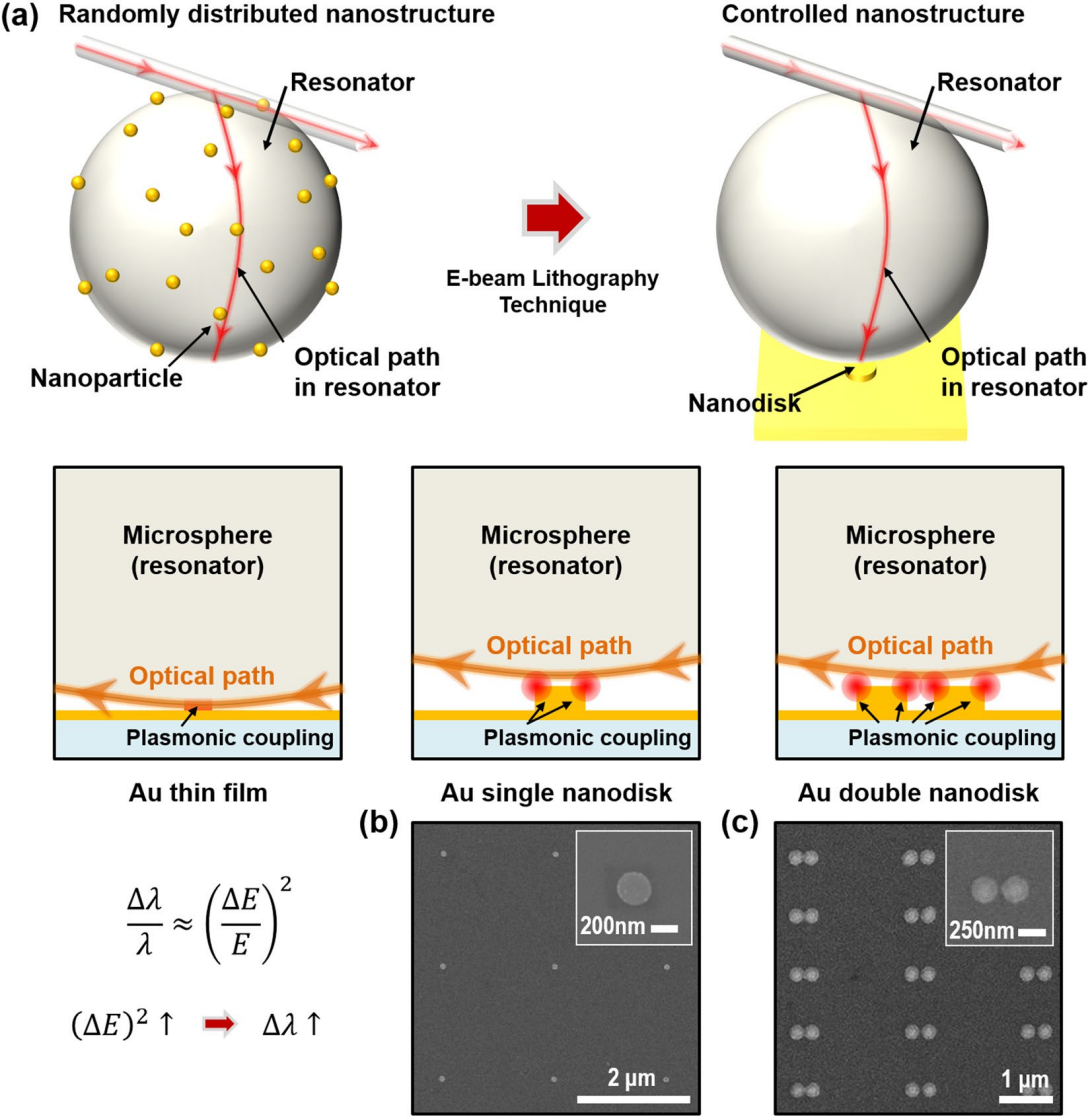
Metallic nanodisk array for plasmonic coupling in whispering gallery mode sensor. (**a**) Schematic illustration of the nanodisk-equipped whispering gallery mode sensor for controlled plasmonic coupling. E-beam lithography technique enables the fabrication of precisely defined nanostructure, including Au thinfilm, single nanodisk and double nanodisk. The position of the microsphere on nanodisk array is controlled by piezoelectric actuators. (**b**,**c**) Scanning electron microscope images of the single nanodisk array and double nanodisk array. The diameter and thickness of each nanodisk were chosen as 300 nm and 50 nm.

A concept of the plamon-coupled WGM biosensor was illustrated in Fig. 1(a). We tested for the fraction of WGM fields that are localized upon coupling to the plasmonic nanodisk structure by observing WGM resonance wavelength shifts as a function of coupling and decoupling to the antenna. The incident light with λ = 1310 nm was circulated inside a microsphere resonator based on total internal reflection, and spatial positions of the resonator were minutely controlled using a mechanical piezo-stage for precisely coupling and decoupling to the nanodisk. Here, TM-polarized light was used to induce the plasmonic phenomenon on the nanodisk defined at thick gold film. Figure 1(b) and (c) show SEM images of fabricated plasmonic nanostructures including single and double nanodisk arrays, respectively. The arrays were deliberately fabricated on 5-nm-thick gold film by electron beam lithography. The array period was 5 µm to exclude any coupling to neighboring disks and to touch the only single nanodisk to a 150- to 200-µm-diameter microsphere resonator. The nanodisk diameter and thickness were chosen as 300 nm and 50 nm, respectively. A previous study suggested that the effective localization of electromagnetic near-fields can be occurred at this nanostructure geometry^35^. Furthermore, 300 nm-sized patterns have been well established to achieve high yields using E-beam lithography and lift-off process^36^.

### Polarization Effect of plasmonic coupling on WGM resonance

**Figure 2:**
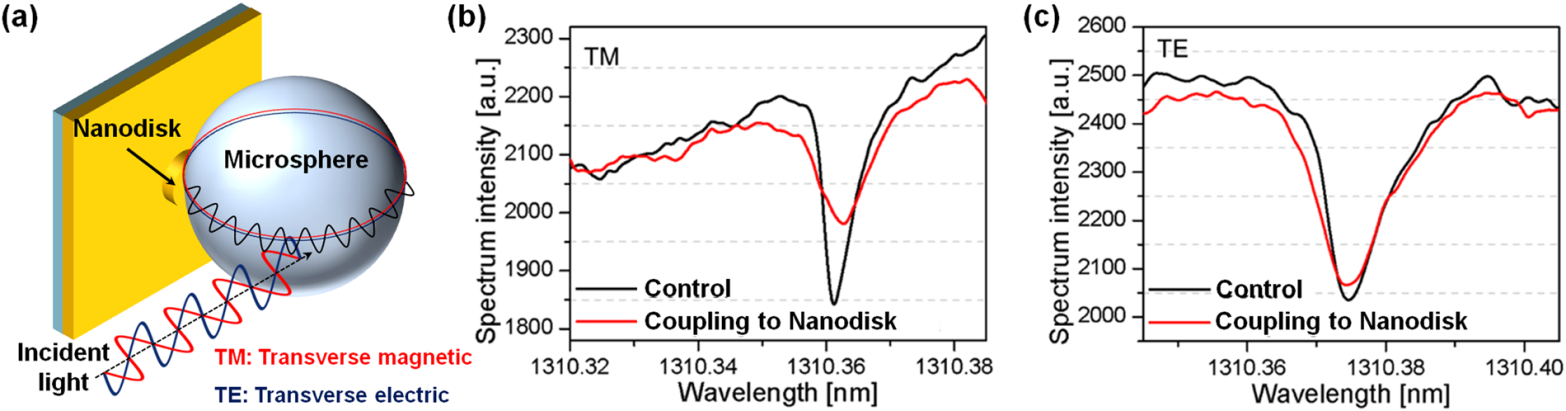
Whispering gallery mode (WGM) resonance of microsphere cavity coupled to the single nanodisk. (**a**) Schematic illustration of the WGM coupling between microsphere and single nanodisk when we shined the incident light with TM and TE polarization. A photodetector connected to the optical fiber recorded the WGM transmission spectrum in real-time. The changes in spectrum intensity of WGM resonance under the incident light with (**b**) TM and (**c**) TE polarization.

The perturbation of a WGM resonance wavelength ∆λ/λ is directly related to the WGM energy fraction ∆E/E localized at the nanoantenna structure that is optically coupled to the microcavity, i.e., ∆λ/λ ≈ (∆**E**/**E**)^2^. Prior to theoretical and experimental investigation, we investigated the effect of WGM polarization with regard to the coupled energy fraction that can indeed be confirmed from WGM resonance shifts. Figure 2(a) shows a schematic diagram to determine the polarization of incident light to optimize the experimental shifts of WGM resonance. We repetitively coupled a 200-µm-diameter microcavity to a 50-nm-height single nanodisk structure fabricated on top of 5-nm-thick gold film and monitored WGM resonance wavelength shifts upon coupling at ~1310 nm wavelengths as shown in Fig. 2(a). Experimentally recorded spectrum intensities before and after the microcavity coupled to the nanodisk at TM and TE polarization were presented in Fig. 2(b) and (c), respectively. A red shift at ~1310 nm wavelength was only observed for TM-polarized light in Fig. 2(b). In contrast, since TE-polarized WGMs do not localize as well as strongly enhance the field on gold nanostructured substrate, the blue shift observed in Fig. 2(c) indicated an overall reduction of the WGM field at TE polarization. To clarify the spatial extend of field localization by incident polarization, we confirmed near-field distributions between the microcavity and the nanodisk by numerical simulations based on a FDTD method (see Supporting Information Figure S2). We confirmed that it was important to control the polarization of WGM for coupling a large energy fraction to the nanodisk, whereas the particular choice of WGM wavelength at 1310 nm had less influence on the coupled energy fraction.

### Spectra Characteristics of plasmon-coupled WGM resonator

**Figure 3:**
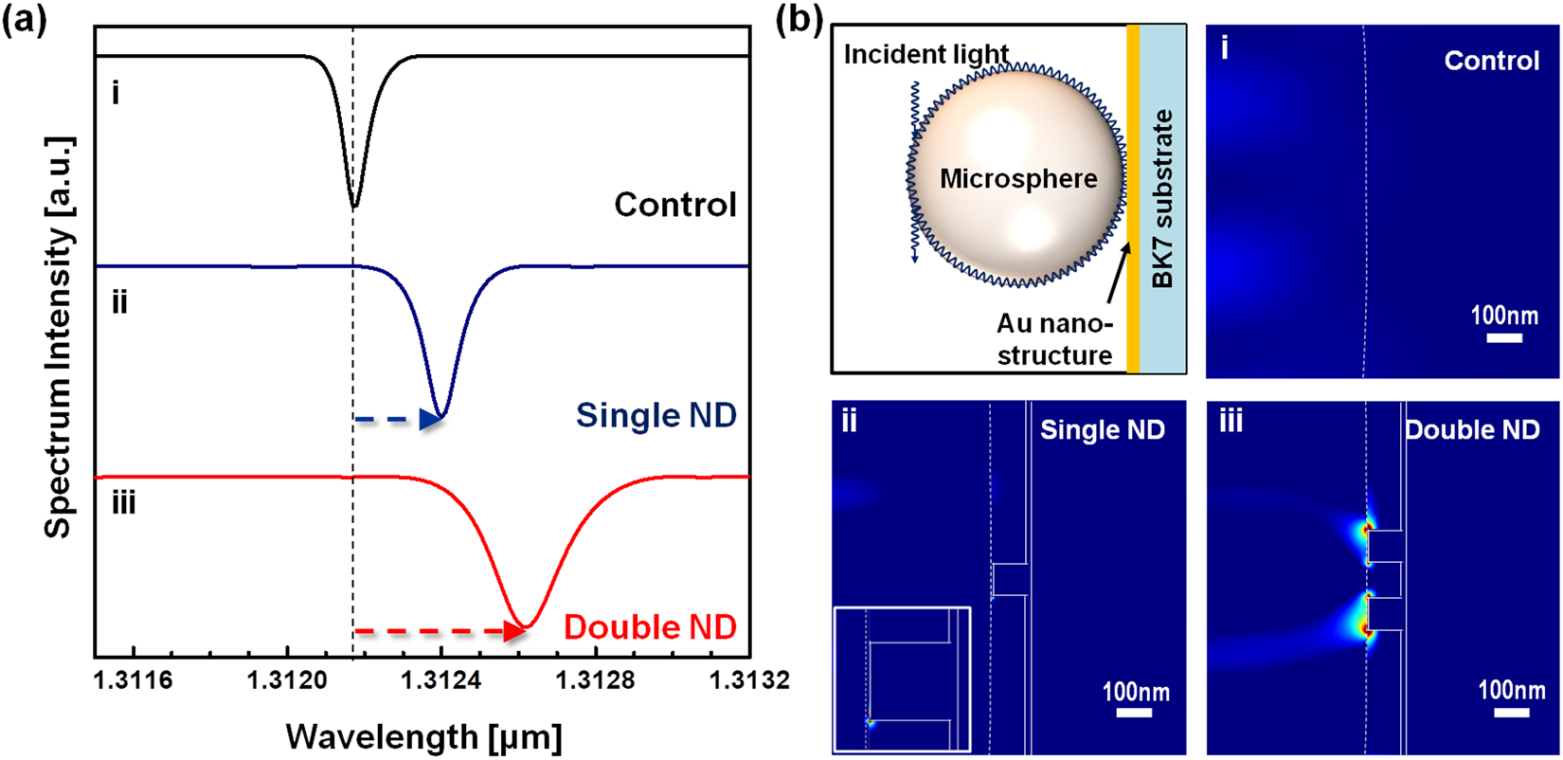
Numerical calculation of plasmonic coupling in WGM sensor mounted on the single and double metallic nanodisks (ND). (**a**) The WGM spectrum intensity of the circulated light in microsphere mounted on thin film and single nanodisk and double nanodisk when the incident light with TM polarization was applied. (**b**) Electric field distribution |E|^2^ in the vicinity of the (i) thinfilm, (ii) single nanodisk and (iii) double nanodisk.

Here, theoretical calculations were performed to investigate the coupled energy fractions when a microcavity coupled to two types of plasmonic nanostructure substrates: single nanodisk and double nanodisks comparing with no plasmonic coupling state as control. Figure 3(a) shows normalized WGM transmission spectra for a 50-µm-diameter microsphere sensor coupled to different plasmonic nanostructures: microsphere in the air for control data (black), coupled to a single nanodisk (blue) and a double nanodisk (red). For both cases of plasmonic coupling, we observed that significant red shifts to longer wavelength were occurred: ∆λ = 0.226 nm for the single nanodisk and ∆λ = 0.446 nm for the double nanodisk. We furthermore found that each perturbation of WGM wavelengths was significantly related with the energy of WGM coupled and localized at the gold nanodisk site. Figure 3(b) shows electric field distributions at the WGM resonance wavelength when a microsphere resonator was perfectly attached to the single nanodisk (ii) and the double nanodisk (iii), compared to the control (i, no plasmonic coupling). Highly localized spots within the region of evanescent waves formed at the surface of the microsphere resonator were created at edges of the nanodisk ridge. Note that much stronger electric field spots were observed in vicinity of a double nanodisk structure, leading to the large red-shift: (∆λ/λ)single = 1.72 × 10^−4^, (∆λ/λ)double = 3.40 × 10^−4^. Even though the absolute value from the simulation do not allow to directly match to experimental results due to variation of the cavity size, the simulation results determine the degree of WGM resonance shift comparing double nanodisk to single nanodisk. We note that a relatively lower Q-factor at the plasmonic nanostructure in the simulation results does not have any bearing on the magnitude of simulated wavelength shifts, and previous works confirms that such scaled simulation models can provide a physical picture of the interactions between the evanescent fields of WGMs and nanostructures^19^. To clarify the spatial extend of field localization, we simulated the spatial and intensity distributions of near-fields at a single nanodisk and a double nanodisk on a microcavity with the diameter ranging from 10 µm up to 100 µm, which can be more directly compared to experimental results. Near-field localization was also verified at the surface of 10-, 50-, and 100-µmdiameter microsphere resonator at a predetermined wavelength of 1310 nm (see Supporting Information Figure S3). Spatial field localization was also observed at the 1310 nm wavelength which substantially deviated from the resonance wavelength. However, differences were observed in field magnitude integrated over the extent of the localized field, compare to the choice of WGM resonance wavelength as shown in Fig. 3. We can clearly identify optical field localization near the single and double nanodisks, indicating strongly localized WGM fields outside of the cavity (see (b), (c), (e), (f), (h) and (i) of Supporting Information Figure S3). With the 10-µm-diameter microcavity, we verified field intensity enhancement due to near-field hotspots localized at the structures by approximately 14- folds and 18-folds at the single and double nanodisks, respectively, over field intensity with no substrate. For a 50- and 100-µm-diameter microcavity, coupling to the double nanodisk has approximately 1.6-folds and 6.7-folds enhancements over coupling to the single nanodisk, respectively. Note that the number of nanodisks can provide enhancement of electrical coupling. But, the intensity of coupling cannot be increased over triple nanodisks, because the antenna effect has a key role of highly enhanced electrical coupling.

### Experimental Results for wavelength spectra of WGM resonators coupled to nanoantenna

**Figure 4:**
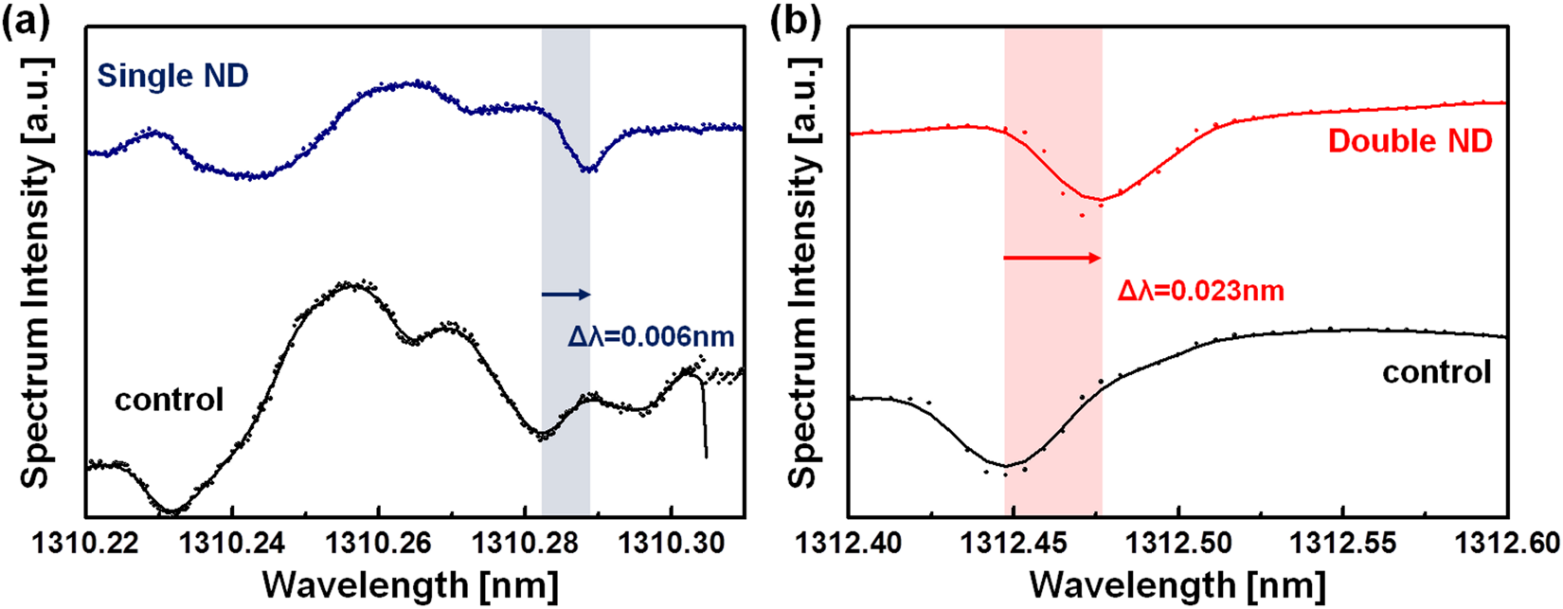
The wavelength shift in the spectrum of WGM sensor on the metallic nanodisk arrays. (**a**) Single nanodisk. (**b**) Double nanodisk.

Figure 4 shows experimental validation studies of spectral resonance shifts due to coupling of WGMs to array of plasmonic nanostructures. We monitored WGM resonance wavelength shifts upon coupling to the single and double nanodisk arrays at ~1310 nm wavelengths. A 200-µm-diameter microcavity was repetitively coupled to the 50-nm-height nanodisk arrays, which was fabricated on top of a BK7 substrate with 2-nm-thick chromium and 5-nm-thick gold adhesion layers. Plasmonic effects of single and double nanodisks were individually measured at TM polarization of the incident light. The wavelength spectrum displayed in Fig. 4(a) and (b) qualitatively reproduces the results of our simulations shown in Fig. 3 and Supporting Information Figure S4]. By comparison with WGM resonance shifts by a single nanodisk, much larger energy fraction of TM-polarized fields on a microcavity was observed by the double nanodisk as presented in Fig. 4. The fractional wavelength shift upon coupling to the double nanodisk was indeed larger than the shift upon coupling to a single nanodisk: (∆λ/λ)_single_ = 1.75 × 10^−4^ and (∆λ/λ)_double_ = 3.43 × 10^−4^, where ∆λ_single_ = 0.23 nm and ∆λ_double_ = 0.45 nm, in a wavelength range of 1310 nm, indicating that the energy fraction of TM-polarized WGM fields is highly localized at the double nanodisk rather than the single nanodisk (see Supporting Information Figure S4).

**Figure 5:**
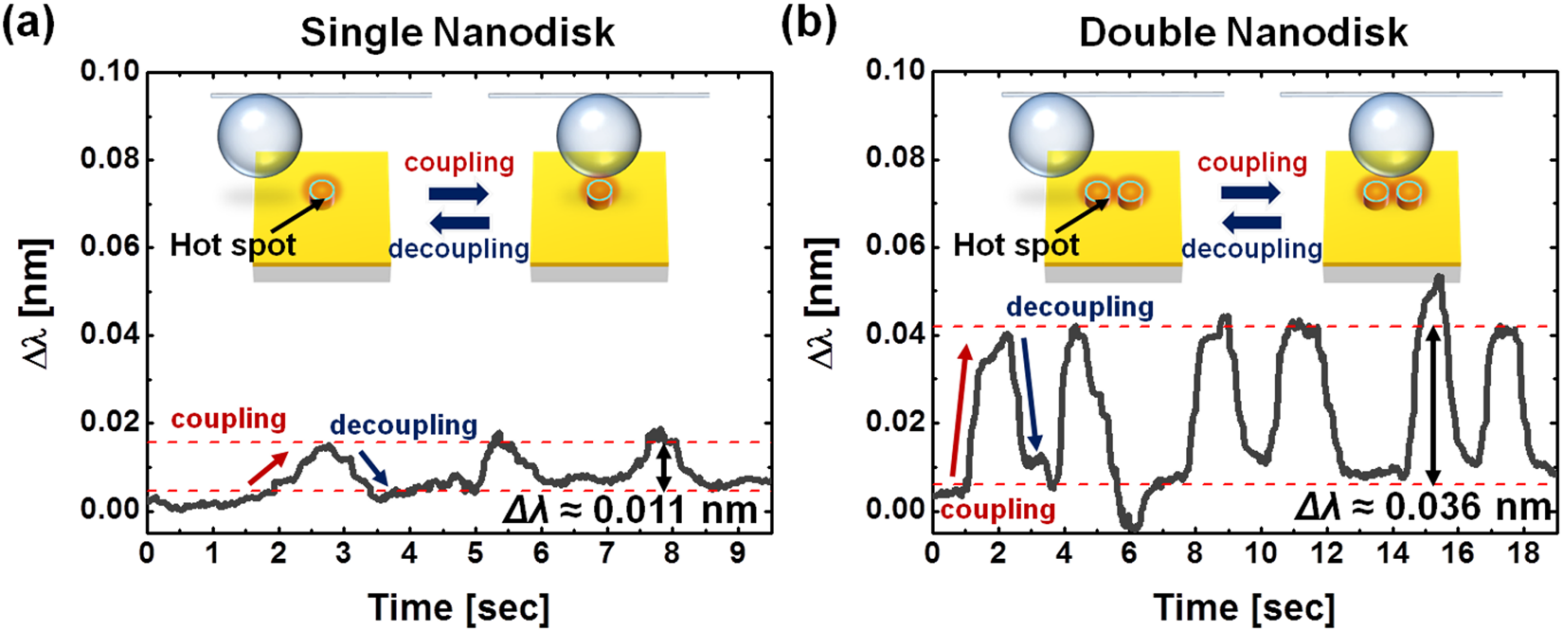
Experimental measurements of WGM resonance wavelength shifts due to coupling of the ~200 μm microsphere to gold film on BK7 glass substrate with and without nanodisk arrays: (**a**) single nanodisk and (**b**) double nanodisks. The WGM resonance wavelengths are near 1310 nm. Arrows indicate optical coupling and decoupling of the microsphere as its vertical position is changed by ~100 nm piezo-stage movements over time.

In addition, we scanned structured surface using a microcavity controlled by a nanostage to measure coupling to nanodisks. When a microcavity laterally scans over a plasmonic nanodisk, the microcavity can act effectively as a probe of the near-field scanning cavity that keeps a fixed distance axially by aligning nanodisks under investigation. Figure 5(a) and (b) show wavelength shifts on coupling or decoupling of single and double nanodisks, respectively. The wavelength shift (∆λ) was measured at 0.036 nm on average as scanning near-fields of the double nanodisk (see Fig. 5(b)), whereas ∆λ on the single disk was 0.011 nm on average (see Fig. 5(a)). More than three times enhancement of resonance shifts was detected by scanning of a WGM microcavity with picometerscale sensitivity, which leads to a very good agreement with far-field results shown in Figs 3 and 4. In other words, far-field wavelength shifts of the WGM resonance tend to be in accordance with the enhancement of localized near-fields.

For biosensing applications, the field localization and amplification has suggested the sensitivity enhancement to detect single molecule^37,38^. Note that the high field intensity provides the significant enhancement of sensor sensitivity even though we observed the degradation of Q-factor by coupling to both single and double nanodisks. The Q-spoiling often observed in previous coupling studies^39,40^. Therefore, high sensitivity can be also achieved in WGM biosensing by a good spatial overlap between highly localized near-field and bound analyte molecules. In addition, this mechanism of WGM nanodisk mediated sensitivity enhancement by efficient spatial field overlap is similar to the chemical enhancement in surface enhanced Raman spectroscopies (SERS). Localizing fields in nanodisks by controlled coupling to WGM biosensors opens up a new route towards single molecule detection if localized field strength (coupled energy fraction) as well as spatial overlap with analyte (co-localization efficiency) is maximized. With more judicious design of nanodisk structures for field localization, for example using bowties^41^, nanopyramids^42^ or concentric necklace nanolenses^43^, further increase in sensitivity of the detection method can be achieved. Further work will focus on optimizing energy fraction with varied designs of nanostructures as well as maximizing overlap efficiency for detecting analyte molecules bound to the nanostructure at extremely low concentration, possible in the single molecule regime.

## Conclusion

In this paper, we have explored the approach to enhance the sensitivity in WGM biosensing using nanodisk arrays fabricated on a planar substrate. We first investigated the effect of WGM polarization with regard to the coupled energy fraction from WGM resonance shifts in experiments, and a resonance wavelength shift was confirmed only for TM-polarized light. Then, calculated near field distributions indicate that the energy fraction of the microcavity field was highly localizes by the existence of the nanodisks at TM polarization. The energy fractions on single and double nanodisks were estimated in calculation and experiment, and we confirmed that the extent of the energy fraction was more drastic on double nanodisk. The wavelength shift upon coupling to the double nanodisk was measured around 4-times larger than to the single nanodisk, indicating a good agreement with simulated results. This plasmon coupling approach gives significant sensitivity enhancements even though Q-factor may be degraded by optical loss. For instance, when a single 100 nm-diameter silica particle adsorbs on cavity, the sensitivity (Δλ/λ) with single and double nanodisks can be obtained 1.36 × 10^−11^, 4.60 × 10^−11^, respectively, whereas the sensitivity for bare WGM system is 3.46 × 10^−12^ ^1,44^.

## Method

### Numerical methods

All modelling results are performed using the finite element method (FEM) to calculate the far-field and near-field optical characteristics in the vicinity of the nanodisk arrays. In calculation, we only model a single unit cell of the 2D nanodisk array, applying periodic boundary conditions to the both vertical sides of the cell. The lower boundary of the simulation domain represents the truncation of the optically thick BK7 glass substrate. The nanodisk with the diameter of 100 nm and the height of 100 nm was built on the Au thin film (10 nm)/BK 7 glass, and the distance between nanodisks was chosen as 100 nm for double nanodisks. Microspheres with diameter from 10 µm to 100 µm were mounted on the nanodisk arrays, and then the incident light with λ = 1310 nm was circulated counterclockwise inside the microcavity. The FEM calculations were carried out based on the frequency domain wave equation,

2 $$\nabla \times (\frac{1}{{\mu }_{r}}\nabla \times {\boldsymbol{E}})-{{\boldsymbol{k}}}_{0}^{2}{\varepsilon }_{r}{\boldsymbol{E}}=0$$

where E is the electric field vector, k_0_ = 2π/λ is the incident wave number, the ε_r_ and µ_r_ are relative permittivity and permeability values, respectively. The whole calculation area is divided into small sub-unit fragments with the mesh grid size of 10 nm. The permittivity of gold is described by interpolated experimental values^45^, whereas the permittivity of BK 7 glass and microsphere assumes the constant value ε_glass_ = 2.26 and ε_microsphere_ = 2.15, respectively.

### Optical set-up

WGMs were excited using tunable distributed feedback (DFB) laser diodes (HSL-20, Santec) operating at the wavelength of 1310 nm. We tested for the energy fraction of WGM field that is localized upon coupling to nanodisk arrays by observing WGM resonance wavelength shifts as a function of coupling and decoupling to the nanodisk. An InGaAs photodetector connected to the optical fiber records the WGM transmission spectrum in real-time as the DFB laser wavelength is rapidly scanned. WGMs are thereby excited at either TE or TM polarization by controlling the polarization in the tapered optical fiber using an in-line optical fiber polarizer^46^. The microspheres.

### Nanodisk arrays fabrication

The nanodisk arrays were fabricated by a standard evaporation method and subsequent electron beam (E-beam) lithography processes. The substrates were prepared by depositing 2 nm of chromium and 10 nm of gold on the BK7 slide glass. The patterns for fabricating the single and double nanodisk arrays were defined using a 300 nm of polymethyl methacrylate (PMMA) photoresist layer on the gold-coated substrate through the E-beam lithography technique. Those patterns were transferred to form 50 nm thick gold nanostructures with 300 nm diameter by a lift-off process after gold deposition by thermal evaporation. In fact, we, from the first, expected to confirm stronger plasmon coupling effects on nanodisk arrays since there have been lots of previous reports that double nanostructures provide highly field localization inside a nanogap^47^. Thus, we fabricated single and double nanodisk arrays which have a same sized nanodisk (300 nm diameter and 50 nm thickness) and a 5 µm period.

## Electronic supplementary material

Supporting information
