# Supplementary material for: Plasmon-Coupled Whispering Gallery Modes on Nanodisk Arrays for Signal Enhancements
Source: Sci Rep. 2017 Sep 15;7:11737. doi: 10.1038/s41598-017-12053-8 (PMC5601917; doi:10.1038/s41598-017-12053-8)
Supplement: Supplementary file 1 — Supporting information [file 41598_2017_12053_MOESM1_ESM.doc]

Supporting Information

Plasmon-Coupled Whispering Gallery Modes on Nanodisk Arrays for Signal Enhancements

**Tae Young Kang^1,†^, Wonju Lee^2,†^, Heesang Ahn^1^, Dong-Myeong Shin^3^, Chang-Seok Kim^1,4^, Jin-Woo Oh^5^, Donghyun Kim^2^,*, and Kyujung Kim^1,4^,***

^1^Pusan National University, Department of Cogno-Mechatronics Engineering, Busan, 46241, Republic of Korea

^2^ Yonsei University, School of Electrical and Electronic Engineering, Seoul, 03722, Republic of Korea

^3^ Pusan National University, Research Center for Energy Convergence Technology, Busan, 46241, Republic of Korea

^4^ Pusan National University, Department of Optics and Mechatronics Engineering, Busan, 46241, Republic of Korea

^5^ Pusan National University, Department of Nano Energy Engineering, Busan, 46241, Republic of Korea

*kimd@yonsei.ac.kr, k.kim@pusan.ac.kr

^†^ These authors contributed equally to this work

**Figure S1.**


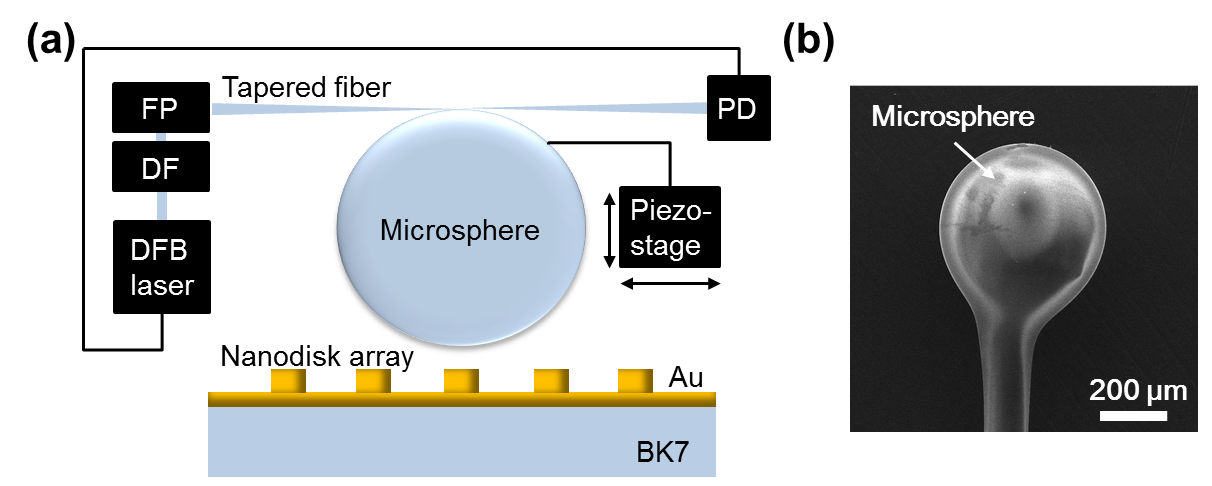


**Figure S1.** (a) Schematic of whispering gallery mode sensor mounted on the metallic nanodisk array. (b) SEM image of microsphere cavity.

**Figure S2.**


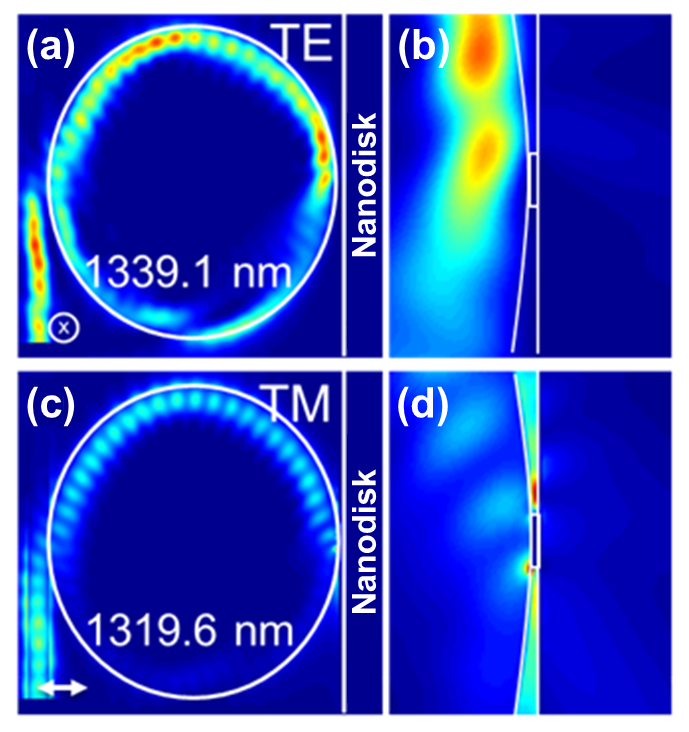


**Figure S2.** **Simulated near-field distribution of a WGM coupled to a single nanodisk.** The microsphere with the diameter of 8μm is excited by a tapered fiber of 500 nm diameter. The nanodisk coupling region is magnified in the right of each panel. (a) Electric field distribution under the incident light with TE polarization (electric field oscillates into the paper plane). (b) Electric field distribution at the boundary between microsphere and nanodisk. (c) Near-field patterns under the incident light with TM polarization (electric field oscillates in parallel to the paper plane). (d) Electric field distribution at the boundary between microsphere and nanodisk.

**Figure S3.**


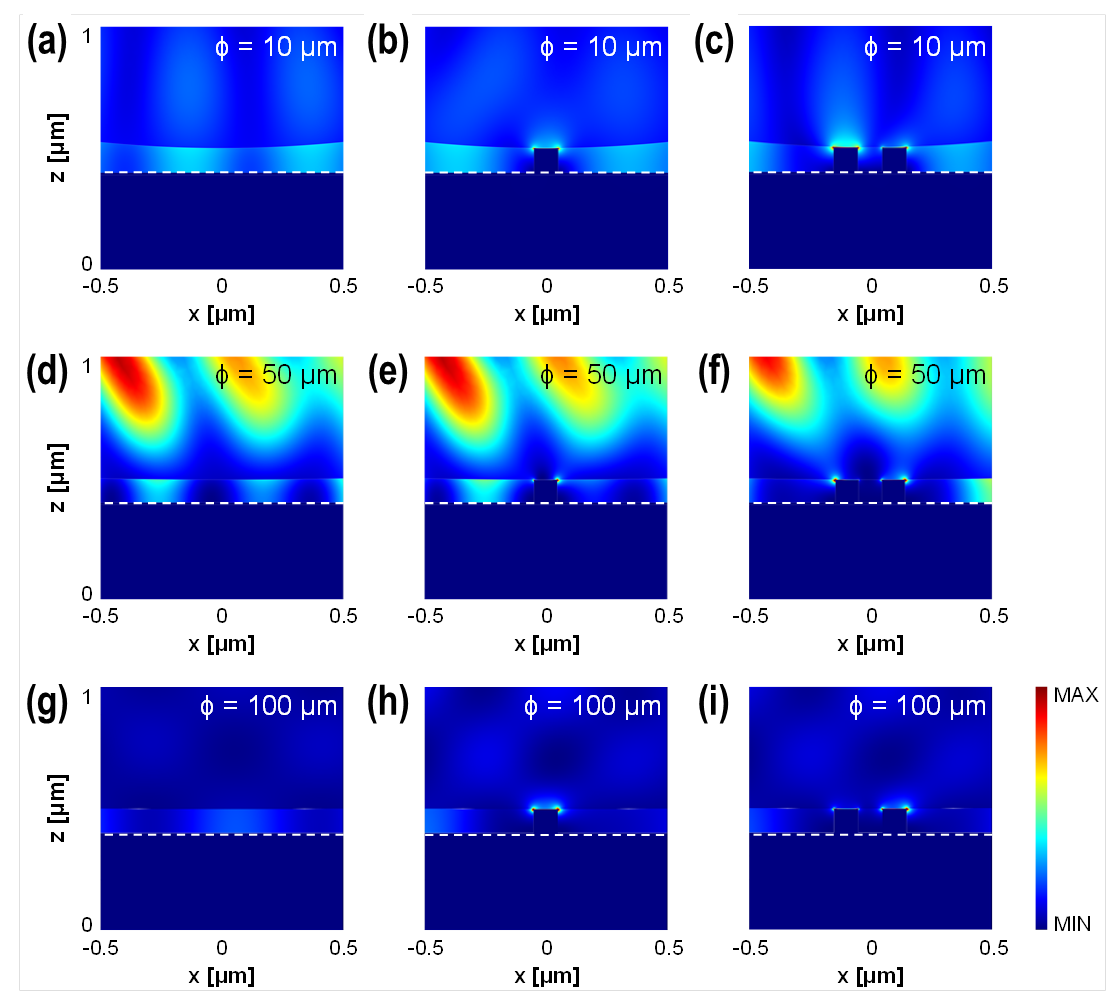


**Figure S3.** **Numerically calculated near-field localization of a WGM cavity coupled to plasmonic nanostructure substrates.** Normalized near-field distribution (|E|2/|E_0_|^2^) at the boundary between microsphere with various diameters and nanodisk. The diameter and thickness of each nanodisk were 100 nm and 100 nm, respectively. The diameter of microsphere and type of nanodisk were chosen as (a) 10 μm and thinfilm, (b) 10 μm and single nanodisk, (c) 10 μm and double nanodisks, (d) 50 μm and thinfilm, (e) 50 μm and single nanodisk, (f) 50 μm and double nanodisk, (g) 100 μm and thinfilm, (h) 100 μm and single nanodisk, (i) 100 μm and double nanodisk.

**Figure S4.**


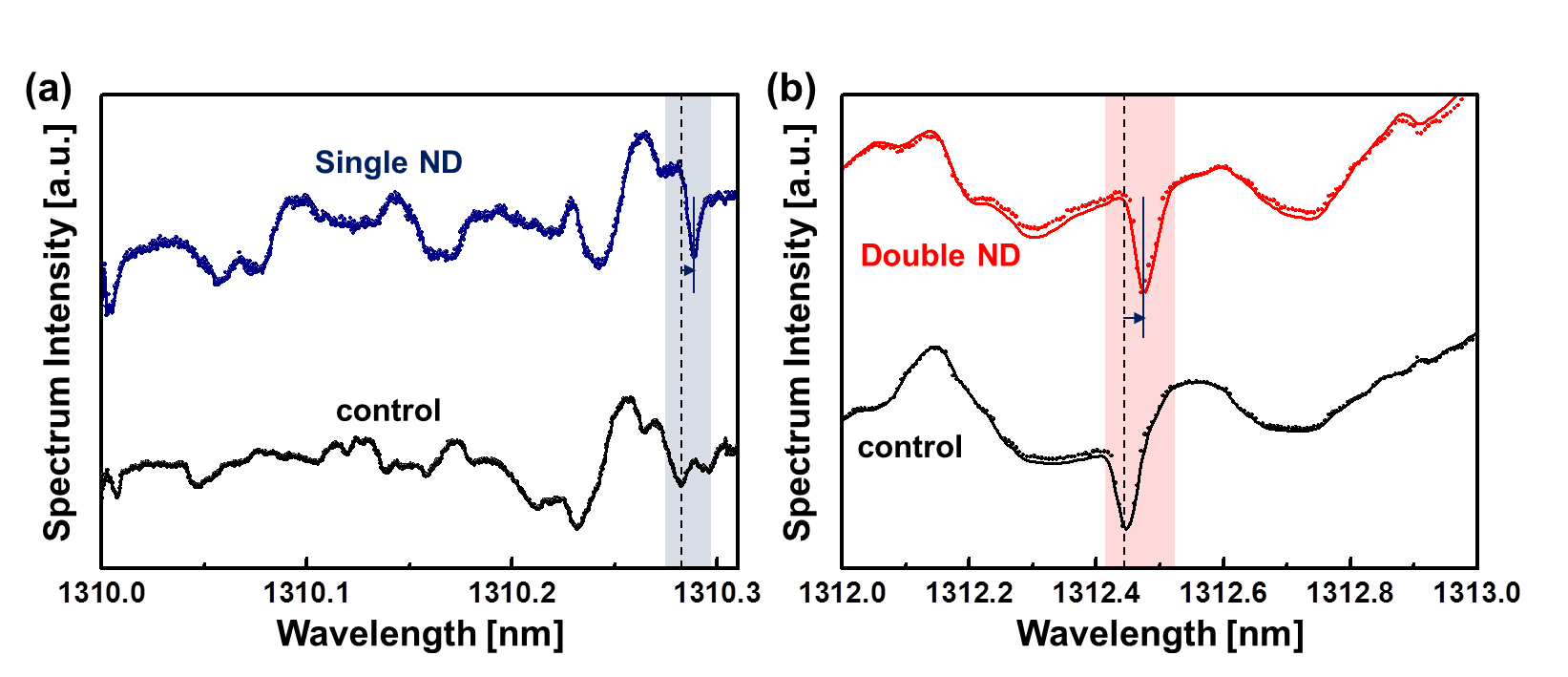


Figure S4. The spectrum of WGM sensor on the metallic nanodisk arrays. (a) Single nanodisk. (B) Double nanodisk.
